# Supplementary material for: Silencing of the Chitin Synthase Gene Is Lethal to the Asian Citrus Psyllid, Diaphorina citri
Source: Int J Mol Sci. 2019 Jul 31;20(15):3734. doi: 10.3390/ijms20153734 (PMC6696430; doi:10.3390/ijms20153734)
Supplement: Supplementary file 1 [file ijms-20-03734-s001.pdf]

Table S1. Sequences and relevant information used for phylogenetic analysis of the chitin synthase gene.

**Genes**	**GenBank No**	***Species***
DcCHS		*Diaphorina citri*
ApCHS	XP_003247517.1	*Acyrthosiphon pisum*
AgCHS1	AFJ00066	*Aphis Glycines*
LsCHS1a	AFC61179	*Laodelphax striatellus*
LsCHS1b	AFC61178	*Laodelphax striatellus*
NlCHS1a	AFC61181	*Nilaparvata lugens*
NlCHS1b	AFC61180	*Nilaparvata lugens*
BmCHS	AFB83705	*Bombyx mori*
CfCHS	ACD84882	*Choristoneura fumiferana*
CmCHS1	AJG44538	*Cnaphalocrocis medinalis*
CmCHS2	AJG44539	*Cnaphalocrocis medinalis*
TCiCHS	KR611528	*Toxoptera citricida*
AaCHS1	XP_001662200.1	*Aedes aegypti*
CqCHS1	XP_001866798	*Culex quinquefasciatus*
DmCHS1	NP_524233	*Drosophila melanogaster*
EoCHS1a	ACA50089	*Ectropis obliqua*
EoCHS1b	ACD10553	*Ectropis obliqua*
HaCHS1	AKZ08594	*Helicoverpa armigera*
HaCHS2	AKZ08595	*Helicoverpa armigera*
MbCHS1	ABX56676	*Mamestra brassicae*
McCHS1	AJF93428	*Mamestra configurata*
AmCHS1	XP_395677.4	*Apis mellifera*
AmCHS2	XP_001121152.2	*Apis mellifera*
PoCHS1	AOE23678	*Phthorimaea operculella*
PoCHS2	AIJ50381	*Phthorimaea operculella*
AgaCHS1a	XP_321336.5	*Anopheles gambiae*
AgaCHS1b	XP_321336.4	*Anopheles gambiae*
AgaCHS2	XP_321951	*Anopheles gambiae*
AqCHS1	ABD74441	*Anopheles quadrimaculatus*
LcCHS1	AAG09712	*Lucilia cuprina*
LmCHS1a	ACY38588	*Locusta migratoria manilensis*
LmCHS1b	ACY38589	*Locusta migratoria manilensis*
MsCHS1	AAL38051	*Manduca sexta*
PxCHS1	BAF47974.1	*Plutella xylostella*
SeCHS1	AAZ03545	*Spodoptera exigua*
SeCHS2	ABI96087	*Spodoptera exigua*
TcCHS1a	AAQ55059	*Tribolium castaneum*
TcCHS1b	AAQ55060	*Tribolium castaneum*
AaCHS2	XP_001651163	*Aedes aegypti*
CqCHS2	XP_001864594	*Culex quinquefasciatus*
DmCHS2	NP_524209	*Drosophila melanogaster*

MsCHS2	AAX20091	*Manduca sexta*
OfCHS1	ACB13821	*Ostrinia furnacalis*
OfCHS2	ABB97082	*Ostrinia furnacalis*
SfrCHS2	AAS12599	*Spodoptera frugiperda*
TcCHS2	AAQ55061	*Tribolium castaneum*
AtCHS	AFM38193	*Anasa tristis*
BdCHS1a	AEN03040	*Bactrocera dorsalis*
BdCHS1b	AGB51153	*Bactrocera dorsalis*
BdCHS2	AGC38392	*Bactrocera dorsalis*
